# The challenge and promise of disentangling neurodevelopmental conditions a commentary on Davis et al. (2024)

**DOI:** 10.1111/jcpp.14106

**Published:** 2024-12-21

**Authors:** Joe Bathelt

**Affiliations:** ^1^ Department of Psychology University of Amsterdam Amsterdam The Netherlands

**Keywords:** Neurodevelopmental disorders, autism spectrum disorders, ADHD, nosology

## Abstract

This commentary evaluates the study by Davis et al. on the early behavioural manifestations of autism spectrum condition (ASC) and attention deficit hyperactivity disorder (ADHD) in preschool children. Davis et al. show how children who later receive dual diagnoses exhibit significantly more severe symptoms and greater behavioural challenges compared to their peers. The study's methodological strengths, including its prospective longitudinal design and well‐validated measures, are highlighted. However, the commentary also critiques the reliance on the traditional diagnostic paradigm, advocating for a shift towards data‐driven or dimensional approaches to better capture the complexities of neurodevelopmental conditions. By adopting such methodologies, the commentary suggests improvements in clinical practices through more personalised interventions, thereby advancing our understanding and treatment of ASC and ADHD.

Current research on autism spectrum condition (ASC) and attention deficit hyperactivity disorder (ADHD) underscores the considerable overlap between these diagnostic categories, particularly in shared behavioural traits such as inattention and impulsivity. This overlap poses distinct challenges for accurate diagnosis and effective intervention, especially given the unique behavioural complexities exhibited by children with dual diagnoses of ASC and ADHD. The study by Davis et al. (2025) enriches this dialogue by meticulously examining the behaviours of preschool children who are later diagnosed with these conditions, either singly or in combination. This commentary discusses the implications within the wider context of research on neurodevelopmental conditions.

## Current research in context

ASC and ADHD are distinct diagnostic categories, but they share common origins and similar behavioural traits such as inattention and impulsivity. The frequent cooccurrence of ASC and ADHD presents significant challenges for accurate diagnosis and effective treatment in child psychology and psychiatry. Previous research, largely focused on school‐aged children, has shown that those diagnosed with both ASC and ADHD exhibit a unique set of behavioural difficulties.

The article by Davis et al. (2025) makes an important contribution to this area by examining the behaviours of preschool children who were later diagnosed with both ASC and ADHD, compared to those diagnosed with only one of these conditions or neither. This study features several strengths that facilitate the robust identification of early behavioural differences linked with ASC, ADHD and their comorbidity. Notably, its prospective longitudinal design serves as an excellent framework for investigating behavioural differences free from the biases of retrospective analysis. Second, the authors attempted to recruit a large community sample from several clinics that include families with diverse racial and socioeconomic backgrounds. It is unfortunate that these commendable efforts were undermined by disruptions caused by the COVID‐19 pandemic, but the intention and implementation provide a template for future studies in this area even if they could not be fully realised in this study. Third, the study uses well‐established and age‐appropriate caregiver ratings scales to assess autism, ADHD and internalising/externalising problems, such as the Modified Checklist for Autism in Toddlers (M‐CHAT‐R/F), ADHD Rating Scale, Preschool Version (ADHD‐RS) and Child Behaviour Checklist, Preschool Version (CBCL) respectively. These measures contribute to the translation of this research, as many clinics will already administer the same measures routinely.

The findings indicate that toddlers who later receive a dual diagnosis of ASC and ADHD display significantly more severe symptoms of autism‐related behaviours, greater internalising and externalising symptoms, and higher autism‐related parent support needs, compared to the other groups. These findings not only advance our scientific understanding of ASC and ADHD but also enhance clinical practices by highlighting the importance of early detection. The behavioural differences identified by Davis et al. indicate the potential for developing targeted early intervention strategies. By improving our ability to identify children with comorbid ASC and ADHD at an earlier stage, we can better tailor interventions to meet their specific developmental needs and alleviate some of the associated caregiver strain. This research thus serves as a cornerstone for future investigations and provides immediate implications for clinical practice.

## Critical analysis

The article by Davis et al. represents a meaningful progression in the understanding of neurodevelopmental conditions, particularly in the identification and management of comorbid ASC and ADHD in early childhood. However, it is crucial to consider some limitations highlighted by the current diagnostic framework on which such studies are based. The reliance on these traditional diagnostic categories to define and treat ASC and ADHD may inadvertently obscure the broader spectrum of these conditions, potentially limiting the effectiveness of early interventions.

Firstly, both ASC and ADHD are acknowledged as highly heterogeneous disorders, with a wide range of manifestations that can vary significantly from one individual to another (Lord et al., 2022; Nigg, Sibley, Thapar, & Karalunas, 2020). This heterogeneity implies that while two individuals may share a diagnosis, their symptoms, the severity of those symptoms and their underlying causes can differ dramatically. Consequently, the use of strict diagnostic categories, which often rely on a checklist of behaviours for qualification, might not adequately capture this diversity.

Moreover, the distinction between ASC and ADHD based on behavioural observations alone has become increasingly problematic. Research has demonstrated that many behavioural traits are shared across the two disorders, such as difficulties with social interaction, problems with executive function, and patterns of inattention and impulsivity (Deserno, Bathelt, Groenman, & Geurts, 2022). This overlap can lead to challenges in accurately diagnosing and differentiating between ASC and ADHD, particularly in young children whose developmental patterns are still emerging.

In addition to behavioural overlap, genetic and biological research has not yet found reliable markers that can distinguish between ASC and ADHD (Cortese et al., 2023). While there have been genetic links identified that suggest a biological basis for both conditions, these markers often overlap with those identified in other traits or developmental conditions (Mattheisen et al., 2022). This genetic overlap calls the validity of the behaviourally‐defined diagnostic labels into question, suggesting that our current categories may be too rigid or simplistic to accommodate the complex biological underpinnings of these conditions.

The adherence to such a diagnostic paradigm not only impacts clinical diagnosis but also shapes research directions and outcomes. Studies structured around these predefined categories may miss subtler, yet clinically significant, variations in neurodevelopmental trajectories that do not conform to traditional diagnostic criteria.

## Alternative: Data‐driven grouping

A common justification for maintaining the current diagnostic paradigm lies in its practical value for clinical decision‐making. Advocates believe that these diagnostic categories efficiently guide the allocation of treatment options and resources to those who need them most, functioning as a basic tool for organising patient care. According to this view, modifying diagnostic groups – such as by defining specific traits for a combined ASC and ADHD category – is less about discovering absolute truths and more about refining a process dictated by practical needs. This system assists clinicians in selecting appropriate interventions and support, using a structured yet approximate system of categorisation.

However, while these practical considerations are a reality of clinical work, it is debatable whether existing diagnostic categories effectively serve as the optimal foundation for creating more precise groupings. Although recognising comorbidity between ASC and ADHD adds some specificity, it may not be sufficient. Transdiagnostic research suggests that categorisations based merely on behavioural similarities do not necessarily correspond with established diagnostic labels (Astle et al., 2019). Alternatively, data‐driven methods have successfully identified more precise categories that better predict future mental health risks (Xie, Bathelt, Fasman, Nelson, & Bosquet Enlow, 2022). These data‐driven approaches provide the definitive classifications necessary for clinical decision‐making while ensuring that the groups are meaningfully distinct.

## Alternative: Dimensional accounts

As healthcare increasingly adopts personalised approaches to treatment, focusing on the distinct dimensions of behavioural difficulties offers a promising route that aligns more closely with the underlying psychological and biological complexities of behavioural traits. This approach is particularly relevant in the context of ASC and ADHD, where specific behavioural dimensions can be distinguished and addressed.

For example, within ASC, behaviours such as social communication and interaction, and repetitive behaviours and restricted interests constitute identifiable dimensions (Happé & Ronald, 2008). Similarly, in ADHD, hyperactivity/impulsivity and inattention emerge as separable dimensions, as evidenced by the corresponding diagnostic subtypes. These dimensions represent more than mere symptoms; they encapsulate the nuanced ways in which these disorders manifest in individuals.

By focusing on these separable dimensions rather than a broad diagnostic label, clinicians can identify early signs of difficulties more accurately. This method allows for the development of more tailored interventions that directly address the specific challenges an individual faces. For instance, a child showing early signs of inattention might benefit from targeted cognitive and behavioural strategies to enhance attention, independent of other ADHD‐related behaviours like hyperactivity.

Such a dimensional approach does not merely treat the symptoms as they appear in a typical diagnostic cluster but seeks to understand and intervene based on the unique behavioural profile of each child. This method is especially useful for children who exhibit atypical constellations of symptoms that do not fit neatly into one diagnostic category but rather span multiple dimensions.

## Future directions

Moving beyond the established diagnostic categories in research on neurodevelopmental conditions present formidable challenges but also immense potential. Both data‐driven grouping and dimensional approaches necessitate large, representative samples and extensive phenotyping, which involves detailed mapping and analysis of an individual's observable traits and behaviours.

Moreover, the importance of tracking developmental trajectories over time cannot be understated. Studies like the one conducted by Davis et al. highlight the need to monitor behavioural changes as children grow to pinpoint early markers of disorders and understand how symptoms evolve. This longitudinal approach is crucial for developing effective interventions but is also resource‐intensive and associated with significant obstacles such as high dropout rates, which can skew data and reduce the reliability of findings.

Furthermore, there is a notable challenge in including diverse populations in these studies. Research often underrepresents individuals from the Global Majority and those from less affluent backgrounds, which can limit the generalisability of findings and potentially overlook important demographic‐specific influences. The study by Davis et al. highlights the challenges of recruiting more inclusive samples, despite following best practices.

Addressing these challenges may require more collaborative efforts to create large, open data resources akin to those used in major initiatives like the Adolescent Brain and Development (ABCD) Study and the HEALthy Brain and Child Development (HBCD) Study. These studies demonstrate the substantial benefits of pooling data and resources across multiple sites, which can significantly enhance the breadth and depth of research findings.

By adopting similar collaborative models for transdiagnostic groups, the field of neurodevelopmental research could see transformative improvements in both understanding and treating conditions like ASC and ADHD. Large‐scale, cooperative research initiatives enable the integration of varied data types and wider participant representation, facilitating a more comprehensive approach to tackling the complexities of neurodevelopmental conditions. This strategy not only addresses the logistical and methodological hurdles but also paves the way for groundbreaking discoveries that could reshape clinical practices and interventions.

## Conclusion

In conclusion, the research conducted by Davis et al. provides a crucial advancement in understanding the early behavioural manifestations of autism and ADHD, especially when these conditions cooccur. This study's findings, which demonstrate more severe symptoms and greater challenges in children with dual diagnoses, underscore the importance of early, accurate identification and intervention. While the study effectively highlights the limitations of current diagnostic frameworks due to the overlapping nature of ASC and ADHD symptoms, it also paves the way for future research to explore more nuanced, data‐driven or dimensional approaches. By moving towards approaches that acknowledge the complexity of neurodevelopmental conditions, we can enhance clinical practices and improve outcomes for affected children and their families. This research not only contributes to our scientific understanding but also offers immediate practical implications for more tailored and effective early intervention strategies.

## Data Availability

Not applicable, this commentary does not include any original analysis of primary data.
